# Comparison of pulmonary vascular permeability index PVPI and global ejection fraction GEF derived from jugular and femoral indicator injection using the PiCCO-2 device: A prospective observational study

**DOI:** 10.1371/journal.pone.0178372

**Published:** 2017-10-17

**Authors:** Wolfgang Huber, Andrea Gruber, Maximilian Eckmann, Felicia Elkmann, Ines Klein, Tobias Lahmer, Ulrich Mayr, Raphael Schellnegger, Jochen Schneider, Gonzalo Batres-Baires, Lisa Fekecs, Analena Beitz, Helena Berbara, Roland Schmid, Alexander Herner

**Affiliations:** Medizinische Klinik und Poliklinik, Klinikum rechts der Isar der Technischen Universität München, München, Germany; Duke University, UNITED STATES

## Abstract

**Background:**

Transpulmonary thermodilution (TPTD) is used to derive cardiac output CO, global end-diastolic volume GEDV and extravascular lung water EVLW. To facilitate interpretation of these data, several ratios have been developed, including pulmonary vascular permeability index (defined as EVLW/(0.25*GEDV)) and global ejection fraction ((4*stroke volume)/GEDV). PVPI and GEF have been associated to the aetiology of pulmonary oedema and systolic cardiac function, respectively. Several studies demonstrated that the use of *femoral* venous access results in a marked overestimation of GEDV. This also falsely reduces PVPI and GEF. One of these studies suggested a correction formula for femoral venous access that markedly reduced the bias for GEDV. Consequently, the last PiCCO-algorithm requires information about the CVC, and correction for femoral access has been shown. However, two recent studies demonstrated inconsistencies of the last PiCCO algorithm using incorrected GEDV for PVPI, but corrected GEDV for GEF. Nevertheless, these studies were based on mathematical analyses of data displayed in a total of 15 patients equipped with only a *femoral*, but not with a *jugular* CVC.

Therefore, this study compared PVPI_fem and GEF_fem derived from femoral TPTD to values derived from jugular indicator injection in 25 patients with both jugular and femoral CVCs.

**Methods:**

54 datasets in 25 patients were recorded. Each dataset consisted of three triplicate TPTDs using the jugular venous access as the gold standard and the femoral access with (PVPI_fem_cor) and without (PVPI_fem_uncor) information about the femoral indicator injection to evaluate, if correction for femoral GEDV pertains to PVPI_fem and GEF_fem.

**Results:**

PVPI_fem_uncor was significantly lower than PVPI_jug (1.48±0.47 vs. 1.84±0.53; p<0.001). Similarly, PVPI_fem_cor was significantly lower than PVPI_jug (1.49±0.46 vs. 1.84±0.53; p<0.001). This is explained by the finding that PVPI_fem_uncor was not different to PVPI_fem_cor (1.48±0.47 vs. 1.49±0.46; n.s.). This clearly suggests that correction for femoral CVC does not pertain to PVPI.

GEF_fem_uncor was significantly lower than GEF_jug (20.6±5.1% vs. 25.0±6.1%; p<0.001). By contrast, GEF_fem_cor was not different to GEF_jug (25.6±5.8% vs. 25.0±6.1%; n.s.). Furthermore, GEF_fem_cor was significantly higher than GEF_fem_uncor (25.6±5.8% vs. 20.6±5.1%; p<0.001). This finding emphasizes that an appropriate correction for femoral CVC is applied to GEF_fem_cor.

The extent of the correction (25.5/20.6; 124%) for GEF and the relation of PVPI_jug/PVPI_fem_uncor (1.84/1.48; 124%) are in the same range as the ratio of GEDVI_fem_uncor/GEDVI_fem_cor (1056ml/m^2^/821mL/m^2^; 129%). This further emphasizes that GEF, but not PVPI is corrected in case of femoral indicator injection.

**Conclusions:**

*Femoral* indicator injection for TPTD results in significantly lower values for PVPI and GEF. While the last PiCCO algorithm appropriately corrects GEF, the correction is not applied to PVPI. Therefore, GEF-values can be used in case of femoral CVC, but PVPI-values are substantially underestimated.

## Background

Transpulmonary thermodilution (TPTD) and pulse contour analysis (PCA) are among the most frequently used techniques of modern haemodynamic monitoring. Both principles have been used separately in the 1^st^ generation COLD-device (TPTD) [1] or the FloTrac and ProAqt-technology (PCA) [2,3]. At least three commercially available devices combine TPTD and PCA (PiCCO [4], EV-1000 [5] and LiDCO [6]). Combination of TPTD and PCA provides *intermittent* calibration of cardiac index CI by TPTD as well as *continuous* assessment of CI, variabilities of the arterial pressure curve such as stroke volume variation (SVV) and pulse pressure variation (PPV). Furthermore, the contractility-index dPmax is continuously derived by PCA. In addition to CI, TPTD provides extravascular lung water EVLW, a marker of pulmonary oedema [1,7–11], and the preload marker global end-diastolic volume GEDV [12–14].

Although an increasing number of parameters are easily and in part continuously derived, the interpretation of numerous parameters is challenging. To facilitate interpretation of these data several ratios (Table 1) have been developed. These ratios are used as a kind of “decision support”, including pulmonary vascular permeability index (defined as EVLW/(0.25*GEDV)), global ejection fraction (defined as (4*stroke volume)/GEDV), cardiac power index (CPI), cardiac function index (CFI).

**Table 1:** Ratios and formulas derived from (transpulmonary) thermodilution and pulse contour analysis.

Pulmonary vascular permeability index (PVPI)	EVLW/(0.25*GEDV)
Global ejection fraction (GEF) [%]	(4*stroke volume)/GEDV
Cardiac function index (CFI) [1/min]	CO/GEDV
Cardiac power index (CPI) [W/m^2^]	MAP*CI*0.00022
Cardiac Power output CPO [W]	MAP*CO*0.0002

CO: Cardiac output

CI: Cardiac Index

EVLW: Extravascular lung water

GEDV: Global end-diastolic volume

MAP: Mean arterial pressure

Several studies demonstrated that these parameters are associated to outcome. Therefore, they might be useful to guide therapy [15–23].

PVPI, GEF and CFI have been associated to the aetiology of pulmonary oedema and systolic cardiac function, respectively. However, a number of studies demonstrated that the use of *femoral* venous access results in a marked overestimation of GEDV [5,24,25]. Overestimation of GEDV also falsely reduces PVPI, GEF and CFI [26,27]. One of these studies suggested a correction formula for *femoral* venous access that markedly reduces the bias compared to *jugular* TPTD derived GEDV_jug [24]. Consequently, the last PiCCO-algorithm requires information about the CVC, and correction for femoral access has been shown. However, two recent studies suggested inconsistencies of the last PiCCO algorithm using uncorrected GEDV for PVPI [26], but corrected GEDV for GEF and CFI [27]. Despite their conclusive results, these studies were based on mathematical analysis of data displayed by the PiCCO in a total of 15 patients equipped with only a *femoral*, but not with a *jugular* CVC.

Therefore, it was the aim of our study to validate the findings of these studies by direct comparison of PVPI_fem and GEF_fem derived from femoral TPTD to values derived from jugular indicator injection in 25 patients with both jugular and femoral CVCs.

## Materials and methods

The institutional review board approved the study (Ethikkommission; Fakultät für Medizin; Technische Universität München 3049/11s). The need for written informed consent from the participants was waived by the review board due to the observational design of the study in accordance with clinical routine in case of different central venous accesses for TPTD-indicator injection. Our institutional SOP for extended haemodynamic monitorings recommends to perform 1–2 additional triplicate TPTDs in case of different CVC-sites used for TPTD to make these measurements better comparable and interpretable. This prospective observational study was conducted in an eight bed general ICU at a university hospital between March 18, 2013 and April 30, 2016. None of the patients has been included in one of the studies or databases previously used for comparison of TPTD-parameters derived from jugular to femoral indicator injection [5,24,26,27].

We prospectively recorded 54 datasets in 25 patients with both jugular and femoral CVC. Each dataset consisted of three triplicate TPTDs with 15ml cold saline solution: The jugular venous access was used as the gold standard TPTD_jug. Two triplicate TPTDs were performed using the femoral access *with* (TPTD_fem_cor) or *without* (TPTD_fem_uncor) information about the femoral indicator injection to evaluate, if correction for femoral GEDV pertains to PVPI_fem and GEF_fem.

To avoid a systematic bias by repeated TPTDs with a total volume of 9*15mL, the three TPTDs were performed in a random order.

The majority (29 out of 54 measurements (54%)) of measurements were performed in patients with both a conventional CVC and a dialysis catheter. In general, CVC and dialysis catheters were inserted in different positions (one in the vena cava superior and the other one in the vena cava inferior). The dialysis catheters were inserted into the femoral and into the jugular vein in 15 (26%) and 14 (28%) of 54 measurements, respectively. In 25 of 54 (46%) measurements two conventional CVCs in opposite positions were used for TPTD.

Indicator injections were performed via a 5-lumen CVC (Multicath 5, Vygon; Aachen, Germany) with a maximum intravascular length of 20 cm and a diameter of 3.15 mm (9.5 Fr) or via a Gambro Gam Cath Dolphin dialysis-catheter (Gambro Gam Cath Dolphin; Gambro Hospal GmbH, Gröbenzell, Germany). Dialysis catheters with a length of 250 mm and a diameter of 13 F were used for femoral access, and catheters with a length of 150–175 mm and a diameter of 13 F were used for jugular RRT-access, respectively. Since the larger volume of the dialysis catheters (up to 1.6ml) might result in a loss of indicator (1.6mL of 15mL, i.e. 11% of the indicator) and in an overestimation of volumetric parameters for the 1^st^ of TPTD-measurement, the dialysis catheters were prefilled with ice cold saline immediately before the 1^st^ indicator injection. The vascular part of the femoral venous catheter was completely inserted under ultrasound guidance. The position of the tip was controlled (and corrected) according to X-ray in case of jugular, but not in case of femoral venous catheter access.

The registration of the arterial TPTD curve was performed as previously described [24,28,29] using a 5-French thermistor-tipped arterial catheter (PV2015L20-A PiCCO catheter; Pulsion Medical Systems SE, Feldkirchen, Germany) with a length of 20cm (5 Fr) placed in the femoral artery and a PiCCO-2-monitor (Pulsion Medical Systems SE, Feldkirchen Gemany).

All PiCCO-2-devices were equipped with the V3.1. algorithm requiring information about the venous catheter site.

### Statistical analyses

All statistical analyses were performed using the IBM SPSS Statistics software version 23 (SPSS Inc., Chicago, IL, USA). The collected data was examined for input data error. Continuous variables are expressed as mean±standard deviation. Categorical variables are expressed as percentages. To compare continuous variables we used Wilcoxon-test for paired samples.

Bland-Altman analysis was used to analyze the agreement between variables derived from jugular and femoral venous catheter sites for both PVPI and GEF as well as to compute the percentage error. The agreement of classification of PVPI (PVPI ≥3; <PVPI<3; PVPI ≤1) derived from different measurements was primarily analyzed using Fisher´s exact test (“agreement yes or no”). Additionally, we calculated kappa-statistics and Kendall´s coefficient of correlation.

To account for different numbers of measurements per patient (range 1–4), analyses were performed for the first measurement in each patient (n = 25; secondary endpoint) in addition to the analysis of all 54 measurements (primary endpoint).

Statistical significance was defined as p<0.05.

## Results

Patients´ characteristics are summarized in Table 2

**Table 2:** 

**Based on individual patients (n = 25)**	
Sex (male:female; n (%))	15:10 (60%:40%)
Age (years±SD)	60±15
Underlying disease (n (%))	
- Sepsis	8 (32%)
- ARDS	4 (16%)
- Cirrhosis/HRS	11 (44%)
- Cardiogenic shock	1 (4%)
- Severe pancreatitis	1 (4%)
Height (cm ± SD)	173±8
Weight (kg ± SD)	79±16
**Based on TPTD measurements (n = 54)**	
Measurements under vasopressors	35/54 (64.8%)
Measurements under mechanical ventilation	46/54 (85.2%)
Measurements under controlled ventilation (CV)	19/54 (35.2%)
Measurements under sinus rhythm (SR)	48/54 (88.9%)
Measurements under SR and CV	19/54 (35.2%)

### Comparison of PVPI-measurements using different indicator injection sites (all measurements)

**Fig 1:**
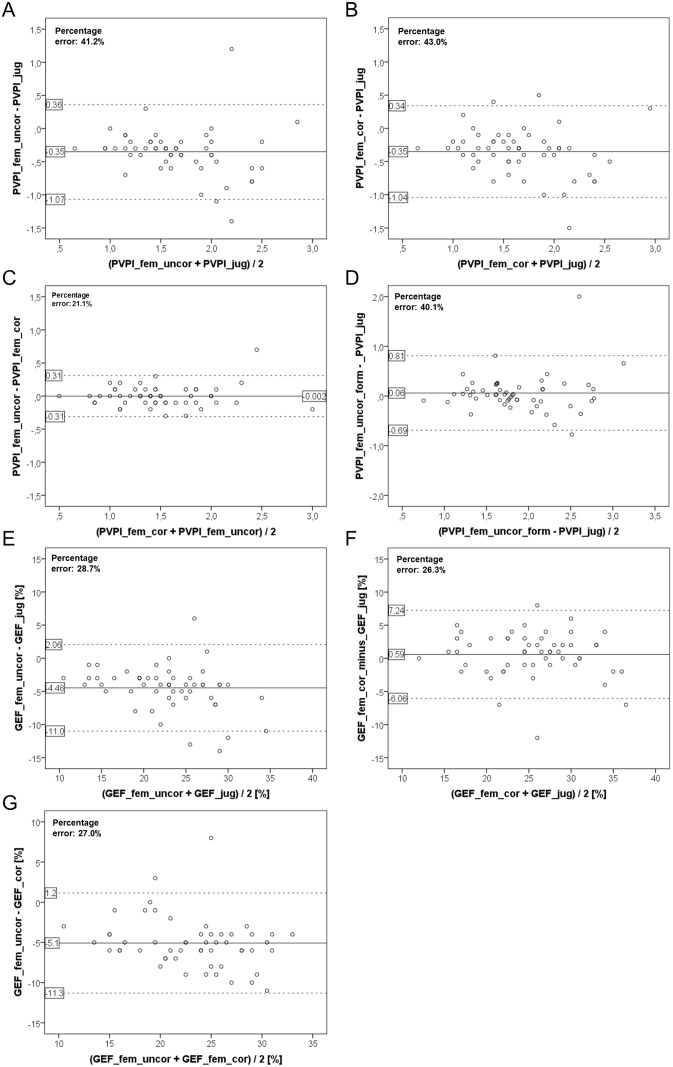
**1A-1F:** Bland Altman plots comparing (1A) pulmonary vascular permeability index PVPI_fem_uncor derived from femoral injection without activating of a potential correction by the device to the gold standard measurement PVPI_jug using a jugular CVC for indicator injection, **(1B)** pulmonary vascular permeability index PVPI_fem_cor derived from femoral injection after activiation of a potential correction by the device to the gold standard measurement PVPI_jug using a jugular CVC for indicator injection, **(1C)** pulmonary vascular permeability index PVPI derived from femoral indicator injection with (PVPI_fem_cor) and without (PVPI_fem_uncor) activation of a potential correction by the device, **(1D)** pulmonary vascular permeability index PVPI derived jugular indicator injection PVPI_jug to PVPI-fem_uncor_form which was derived from femoral indicator injection without activation of a potential correction by the device, but correction based on the formula suggested for correction of femoral indicator injection derived GEDVI: GEDVI_corrected_ [mL / m^2^] = 0.539 * GEDVI_uncorrected_—15.17 + 24.49 * CI_uncorrected_ 2.311* BW_ideal._ PVPI_fem_uncor_form was calculated by multiplying PVPI_fem_uncor with the ratio 0.25*GEDV_uncorrected_/0.25*GEDV_corrected_, **(1E)** global ejection fraction GEF_fem_uncor derived from femoral injection without activating of a potential correction by the device to the gold standard measurement GEF_jug using a jugular CVC for indicator injection, **(1F)** global ejection fraction GEF_fem_cor derived from femoral injection after activiation of a potential correction by the device to the gold standard measurement GEF_jug using a jugular CVC for indicator injection, **(1G)** global ejection fraction GEF derived from femoral indicator injection with (GEF_fem_cor) and without (GEF_fem_uncor) activation of a potential correction by the device.

In the totality of all 54 measurements PVPI_fem_uncor was significantly lower than PVPI_jug (1.48±0.47 vs. 1.84±0.53; p<0.001). This resulted in a bias of -0.35±0.36 and a percentage error of 41.2% (S1 Fig, Fig 1A).

The coefficients of variation (CV) were in the same range for PVPI_jug and PVPI_fem_uncor (29% and 32%, respectively).

Similarly, PVPI_fem_cor was significantly lower than PVPI_jug (1.49±0.46 vs. 1.84±0.53; p<0.001; S1 Fig; Fig 1B) with a bias of -0.35±0.36 and a percentage error of 43.0%.

This is explained by the finding that PVPI_fem_uncor was not different to PVPI_fem_cor (1.48±0.47 vs. 1.49±0.46; p = 0.614.; S1 Fig; Fig 1C) with a bias of -0.002±0.16 and a percentage error of 21.1%. Furthermore, the CV-values were in the same range for PVPI_fem_cor and PVPI_fem_uncor (31% and 32%, respectively).

This strongly suggests that the correction for femoral CVC does not pertain to PVPI.

The relation of PVPI_jug/PVPI_fem_uncor (1.84/1.48; 124%) is in the same range as the ratio of GEDVI_fem_uncor/GEDVI_fem_cor (1056ml/m^2^/821mL/m^2^; 129%).

Therefore, PVPI_fem_form was calculated by correcting PVPI_fem_uncor by multiplication of PVPI_fem_uncor with the ratio GEDVI_fem_uncor/GEDVI_fem_cor using the recently suggested correction formula for GEDVI_fem [24]:

$$GEDVI\_fem\_cor\;\left[mL\;/\;m^{2}\right]\;=0.539\;*\;GEDVI\_fem\_uncor\;-\;15.17\;+\;24.49\;*\;CI\_fem\_uncor\;+\;2.311*\;BW_{ideal}.$$

Consequently, for ex-post-correction of PVPI_fem_uncor we calculated PVPI_fem_uncor_form by multiplying PVPI_fem_uncor with the ratio GEDVI_fem_uncor/GEDVI_fem_cor:

PVPI_fem_uncor_form=PVPI_fem_uncor*(GEDVI_fem_uncor/GEDVI_fem_cor).

PVPI_fem_uncor_form was not significantly different from the gold standard PVPI_jug (1.89±0.57 vs. 1.84±0.53; p = 0.368; S1 Fig; Fig 1D) with a bias of 0.06 and a percentage error of 40.1%. The CV-values were comparable for PVPI_fem_uncor_form and PVPI_jug (30% and 29%, respectively).

To evaluate the impact on the potential clinical decision process we compared the distribution of PVPI-values in accordance with ALI/ARDS (PVPI ≥3) or hydrostatic pulmonary oedema (1<PVPI<3) or outside these classifications (PVPI ≤1) for the “gold-standard” PVPI_jug vs. the classifications according to PVPI_fem_uncor and PVPI_fem_uncor_form, respectively (Table 3). The agreement of PVPI_fem_uncor was 43 out of 54 (79.6%) which was significantly different to the gold standard of PVPI_jug (p<0.001; Fisher´s exact test). The agreement of PVPI_fem_uncor_form was 50/54 (92.6%), which was not significant different to PVPI_jug (p = 0.059).

**Table 3:** Measurements of pulmonary permeability index PVPI_fem_uncor and PVPI_jug categorized “PVPI ≥3”, “1<PVP<3” and “PVPI≤1”. Measurements classified in the same category are depicted in bold letters.

		**PVPI_fem_uncor**			**PVPI_fem_uncor_form**		
		≤1	1<PVPI<3	≥3	≤1	1<PVPI<3	≥3
**PVPI_jug**	≤1	**1** **(1.9%)**	1 (1.9%)	0 (0.0%)	**1** **(1.9%)**	1 (1.9%)	0 (0.0%)
	1<PVPI<3	9 (16.7%)	**42** **(77.8%)**	1 (1.9%)	1 (1.9%)	**49** **(90.7%)**	2 (3.7%)
	≥3	0 (0.0%)	0 (0.0%)	**0** **(0.0%)**	0 (0.0%)	0 (0.0%)	**0** **(0.0%)**

Furthermore, kappa-statistics and Kendall´s coefficient of correlation confirm a significant agreement with PVPI_jug for PVPI_fem_uncor_form (kappa = 0.308; p = 0.002; Kendall´s coefficient of correlation = 0.357; p = 0.009), but not for PVPI_fem_uncor (kappa = 0.100; p = 0.259; Kendall´s coefficient of correlation = 0.156; p = 0.152).

Assuming that GEDVI_cor, but not PVPI_cor is corrected for femoral indicator injection we calculated PVPI_cor_calc based on EVLW_cor and GEDV_cor (GEDV_cor = GEDVI_cor * predicted body surface area (BSA); EVLW_cor = EVLWI_cor * predicted bodyweight; PVPI_cor_calc = EVLW_cor/0.25*GEDV_cor). Interestingly, PVPI_cor_calc was significantly higher than PVPI_cor (1.97±0.50 vs. 1.49±0.46; p<0.001). Although PVPI_cor_calc was also slightly higher compared to PVPI_jug (1.97±0.50 vs. 1.84±0.53; p = 0.006), the amount of the difference PVPI_cor_calc—PVPI_jug was substantially smaller than for the difference PVPI_cor—PVPI_jug (0.13±0.34 vs. 0.35±0.35; p<0.001).

While the bias compared to PVPI_jug was slightly higher for PVPI_cor_calc than for PVPI_uncor_form, the agreement with the previously mentioned three classifications (PVPI ≤1), 1<PVI<3 and PVPI ≥3 of PVPI_jug was 53 out of 54 (98.1%) for PVPI_cor_calc. This distribution was not significant different to PVPI_jug, and the agreement was significantly better than for PVPI_uncor (53 out of 54 vs. 43 out 54; p = 0.004).

Furthermore, kappa-statistics and Kendall´s coefficient of correlation confirm significant agreement of PVPI_fem_cor_calc with PVPI_jug (kappa = 0.486; p<0.001; Kendall´s coefficient of correlation = 0.507; p<0.001).

### Comparison of PVPI-measurements using different indicator injection sites (first measurement in each patient)

To account for different numbers of measurements per patient, we separately analysed the first measurements of each patient (n = 25). In general, these analyses confirmed the primary analyses of all 54 datasets:

PVPI_fem_uncor and PVPI_fem-cor were not significantly different (1.46±0.50 vs. 1.43±0.43; p = 0.498). However, both were significantly lower compared to PVPI_jug (1.73±0.51; p<0.001 for both comparisons). This underlines that in case of femoral CVC an uncorrected GEDVI is used to calculate PVPI, even if the correct information of the femoral CVC-position is given to the device.

### Comparison of GEF-measurements using different indicator injection sites (all measurements)

GEF_fem_uncor was significantly lower than GEF_jug (20.6±5.1% vs. 25.0±6.1%; p<0.001; percentage error 28.7%; S2 Fig; Fig 1E).

By contrast, GEF_fem_cor was not different to GEF_jug (25.6±5.8% vs. 25.0±6.1%; n.s.; percentage error 26.3%; S2 Fig; Fig 1F). Furthermore, GEF_fem_cor was significantly higher than GEF_fem_uncor (25.6±5.8% vs. 20.6±5.1%; p<0.001; percentage error 27.0%; S2 Fig; Fig 1G). These findings emphasize that an appropriate correction for femoral CVC is applied to GEF_fem_cor.

The extent of the correction (25.5/20.6; 124%) for GEF by giving the information of the femoral indicator injection and the relation of PVPI_jug/PVPI_fem_uncor (1.84/1.48; 124%) are in the same range as the ratio of GEDVI_fem_uncor/GEDVI_fem_cor (1056ml/m^2^/822mL/m^2^; 128%). This further suggests that GEF, but not of PVPI is corrected in case of femoral indicator injection.

### Comparison of GEF-measurements using different indicator injection sites (first measurement in each patient)

Also for GEF, the analyses of the first measurement in each patient confirmed the findings of the analyses of all datasets: Despite a slight, but statistically significant overestimation GEF_fem_cor was comparable to GEF_jug (26.4±6.3% vs. 25.2±6.6%; p = 0.043; bias 1.1±2.6%; PE 19.5%). However, both GEF_jug and GEF_fem_cor were significantly higher compared to GEF_fem_uncor (21.1±5.5%; p<0.001 for both comparisons).

## Discussion

Several recent studies suggest a marked overestimation of GEDV(I) and an underestimation of PVPI in case of using a femoral CVC for indicator injection compared to the gold standard of jugular injection. Interestingly, a similar phenomenon was found in case of a misplacement of the subclavian central venous catheter tip into the jugular vein [30]. One of these studies suggested a correction formula for GEDVI derived from femoral indicator injection. This formula is based on GEDVI_fem_uncor, CI_fem_uncor and ideal bodyweight [24].

This formula appropriately corrected GEDVI in a small validation cohort. Furthermore, several studies suggest that a similar formula has been integrated to the last PiCCO-2-algorithm. However, based on mathematical analyses of the data displayed for PVPI and GEF, two recent studies demonstrated that PVPI obviously is not corrected for femoral injection, whereas the values for GEF were in line with a correction for femoral indicator injection [26,27]. Since analyses were performed in small cohorts with only femoral CVCs, the final prove of these results in patients equipped with both jugular and femoral catheters remained to be demonstrated.

Therefore, we performed three triplicate TPTDs in patients with a CVC in the jugular (one triplicate TPTD) and in the femoral vein (two triplicate TPTDs). This setting allowed for comparison of uncorrected and potentially corrected parameters derived from *femoral* CVC to the corresponding values derived from *jugular* indicator injection. Furthermore, to the best of our knowledge this study was the first to compare TPTD parameters derived from femoral indicator injection with and without giving the information about the correct catheter site to the PiCCO-device equipped with the last algorithm requiring information about the catheter site. Using this approach we were also able to analyse, if and to which extent parameters derived from femoral indicator injection are corrected by the device.

The study investigated six key questions:

### 1.) Does the last PiCCO-2 algorithm correct GEDVI derived from femoral indicator injection TPTD?

Regarding this issue, our data demonstrated that GEDVI_fem_uncor was substantially corrected to derive GEDVI_fem_cor which was about 22% lower (mean values of 1056 mL/m^2^ and 822ml/m^2^, respectively).

### 2.) Is the correction of GEDVI_fem applied to PVPI_fem_uncor?

Furthermore, this study showed that PVPI derived from femoral indicator injection markedly underestimates PVPI_jug. Similar underestimation for PVPI_fem_uncor and PVPI_fem_cor as well as the absence of a difference between PVPI_fem_uncor and PVPI_fem_cor suggest that PVPI derived from femoral indicator injection is not corrected at all, irrespective of the information about the catheter site given to the device. This means that the device “ignores” the information about femoral CVC with regard to PVPI. As demonstrated by the distribution of PVPI-values categorized as in line with “ARDS/ALI (PVPI≥3)”, with “hydrostatic pulmonary oedema (1<PVPI<3)” and “outside of these two categories (PVPI≤1)” was substantially different for TPTDs derived from jugular and femoral TPTDs.

### 3.) Is PVPI_fem reproducible, or is femoral indicator injection derived PVPI “instable” per se?

Based on the evidence that PVPI_fem is not corrected at all, the two measurements of PVPI_fem_cor and PVPI_fem_uncor can be compared to analyze accuracy and precision of femoral TPTD-derived PVPI. A low bias of -0.002±0.16, a percentage error of 21.1% and similar CV-values (0.31 and 0.32, respectively) for the comparison of PVPI_fem_cor and PVPI_fem_uncor are in line with a precise and accurate femoral measurement of PVPI when compared to each other.

### 4.) Can PVPI_fem_(un)cor be corrected by the previously suggested formula with an acceptable bias?

Mathematical application of the previously suggested correction formula for GEDVI_fem to PVPI_fem_uncor resulted in an accurate correction as evidenced by the comparison of the mean values of PVPI_jug and PVPI_fem_uncor_form with a low bias of 0.06 as well as by a similar distribution of their clinical relevant categories.

### 5.) Can PVPI_fem_(un)cor be corrected by the previously suggested formula with an acceptable precision?

Since the percentage error has been introduced as a measure of precision of *cardiac index* derived from different methods compared to a gold standard technique, the application of PE and its critical threshold to other parameters has to be done with caution. This applies in particular to the use of the PE for combined formulas such as GEF and PVPI, since imprecisions of the different components might add up. Therefore, the previous study on femoral indicator injection TPTD restricted the application of PE to comparisons of CI and did not report PEs for EVLWI, GEDVI or PVPI [24].

At first glance, higher PE-values between 40.1% and 43.0% for all measurements derived from femoral indicator injection compared to PVPI_jug suggest that correction for PVPI_fem with appropriate precision regarding PVPI_jug might be more complex. This can be explained by the interdependent calculation of EVLW and PBV when using the single indicator TPTD technique. According to this approach an overestimation of GEDV and PBV necessarily results in an underestimation of EVLW, since EVLW is estimated as the difference of pulmonary thermo-volume PTV minus 0.25*GEDV. This interdependent calculation of two components of PVPI (i.e. EVLW and GEDV) might also explain a small number of outliers (see No. 42 and 46 in S1 Fig) and the higher PE-values for PVPI_fem-uncor_form of 40.1% even after ex-post correction by the previously suggested formula, since only GEDV, but not EVLW were corrected by this approach. On the other hand, the small but significant overestimation of EVLWI by a mean of 0.83mL/kg in the above-mentioned study [24] suggests that mathematical underestimation of EVLW might be over-compensated by some kind of indicator-loss due the enlarged thermodilution volume in case of femoral indicator injection.

Finally, based on the data of this study some of these considerations seem to be theoretical, since pragmatic correction of PVPI by the previously suggested correction formula for femoral indicator injection derived GEDVI resulted in a low bias and an appropriate categorization according to clinically relevant thresholds.

### 6.) Is GEF appropriately corrected?

Our data indicate that GEF derived from femoral indicator injection is appropriately corrected by the new PiCCO-2 algorithm. In addition to a low bias for GEF_fem_cor vs. GEF_jug, the percentage error was acceptable with 26.3%. This also applies to the comparison of femoral measurements with and without correction by the device (GEF_fem_cor vs. GEF_fem_uncor) with a percentage error of 27.0%.

### Practical implications

Since recent data did not indicate an increased risk of catheter-related bloodstream infections in case of a *femoral* catheter site, and due to the “ease and perceived lower insertion risk at this site” [31], the femoral venous access remains to be frequently chosen catheter site used in up to a third of CVCs [32,33]. The percentage might be even higher in severely ill patients requiring central venous access for different purposes including extracorporeal organ support [28].

According to this study GEF is appropriately corrected in case of femoral indicator injection and can be used with the same normal ranges as for jugular or subclavian indicator injection.

However, PVPI remains to be uncorrected for femoral vein indicator injection, resulting in a substantial and clinical relevant underestimation of PVPI. This might lead to a substantial number of measurements misclassifying PVPI-values as “hydrostatic oedema” instead of “inflammatory pulmonary oedema”. Appropriate correction of PVPI is of high clinical importance, since PVPI cannot be replaced by other techniques, while echocardiographic ejection fraction gives an information which is comparable to GEF. Consistent correction for femoral indicator injection derived GEDVI, PVPI and GEF is of high practical relevance and would avoid the introduction of separate “normal” ranges for PVPI in case of femoral indicator injection.

As long as consistent correction for GEDVI and PVPI is not given by the PiCCO-algorithm, the clinician can re-calculate PVPI_fem_cor_calc based EVLWI_fem_cor and GEDVI_fem_cor. A previous study demonstrated that not only GEDVI_fem_cor, but also GEDV_fem_cor are corrected in case of the correct information about the femoral indicator injection [27]. Therefore, the clinician can use unindexed values of GEDV_fem_cor and EVLW_fem_uncor as displayed by the device to calculate PVPI_fem_cor_calc. This facilitates the calculation of PVPI_fem_cor_calc, since recalculation of EVLW and GEDV from EVLWI and GEDVI can be avoided.

**Fig 2:**
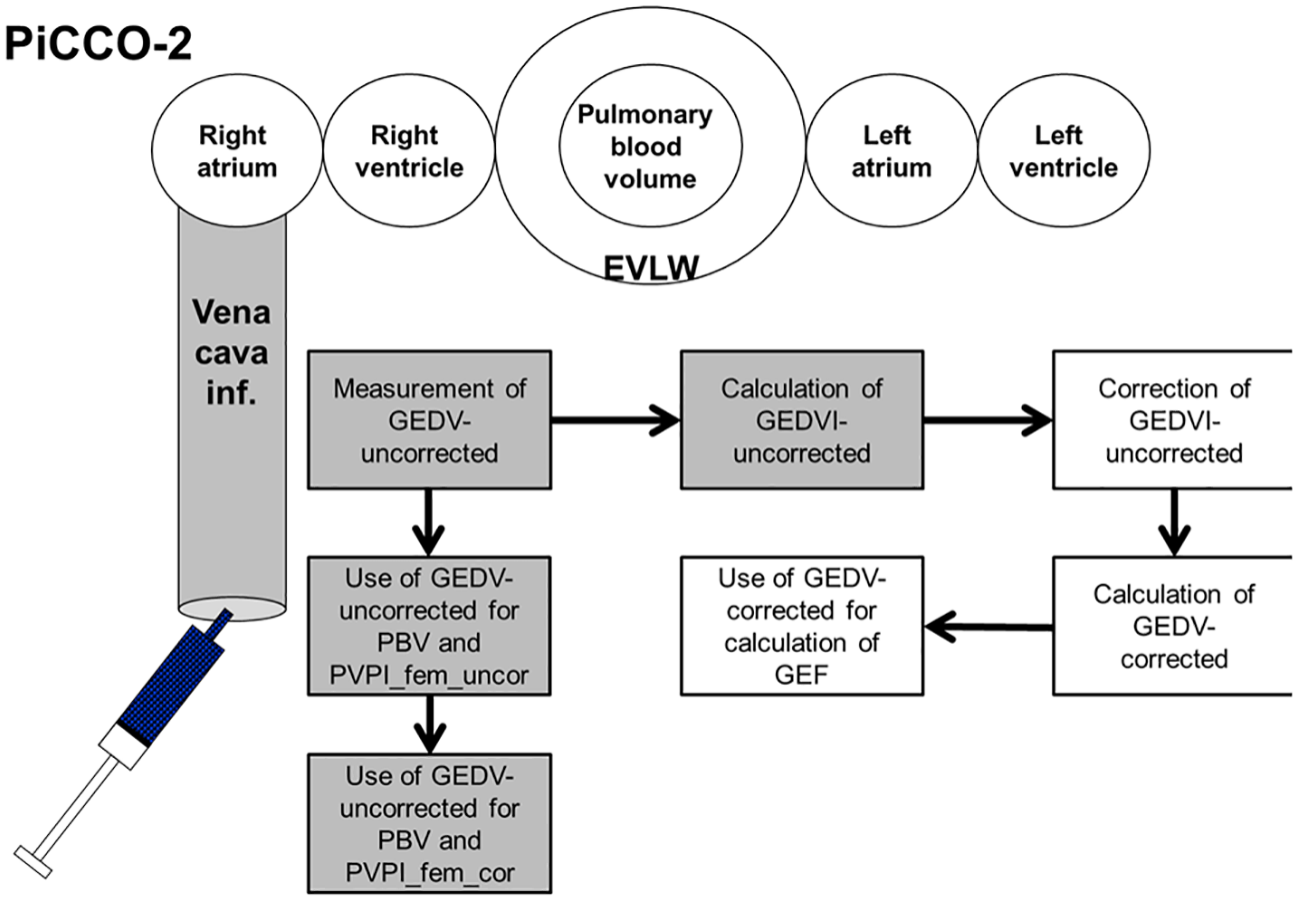
Algorithm for the calculation of GEDV, GEDVI, PBV, PVPI and GEF as displayed by the PiCCO-2 software V 3.1. Confounders (volume of VCI) and confounded values with marked deviation from corresponding measurements using jugular access (PBV, PVPI_fem) are shaded. The primarily measured GEDVfem_uncor is indexed to predicted body surface area (BSA) resulting in GEDVI_fem_uncor. In a next step GEDVI_fem_cor is derived from correction of GEDVI_fem_uncor according to the previously suggested formula. Unindexed GEDV_fem_cor results from multiplication of GEDVI_fem_cor by predicted BSA and is used for calculation of GEF_fem_cor. By contrast, PVPI_fem is based on GEDV_fem_uncor irrespective of giving the information about the femoral CVC-position. GEDV(I): global end-diastolic volume (index) PBV: pulmonary blood volume PVPI: pulmonary vascular permeability index GEF: global ejection fraction BSA: body surface area

**Fig 3:**
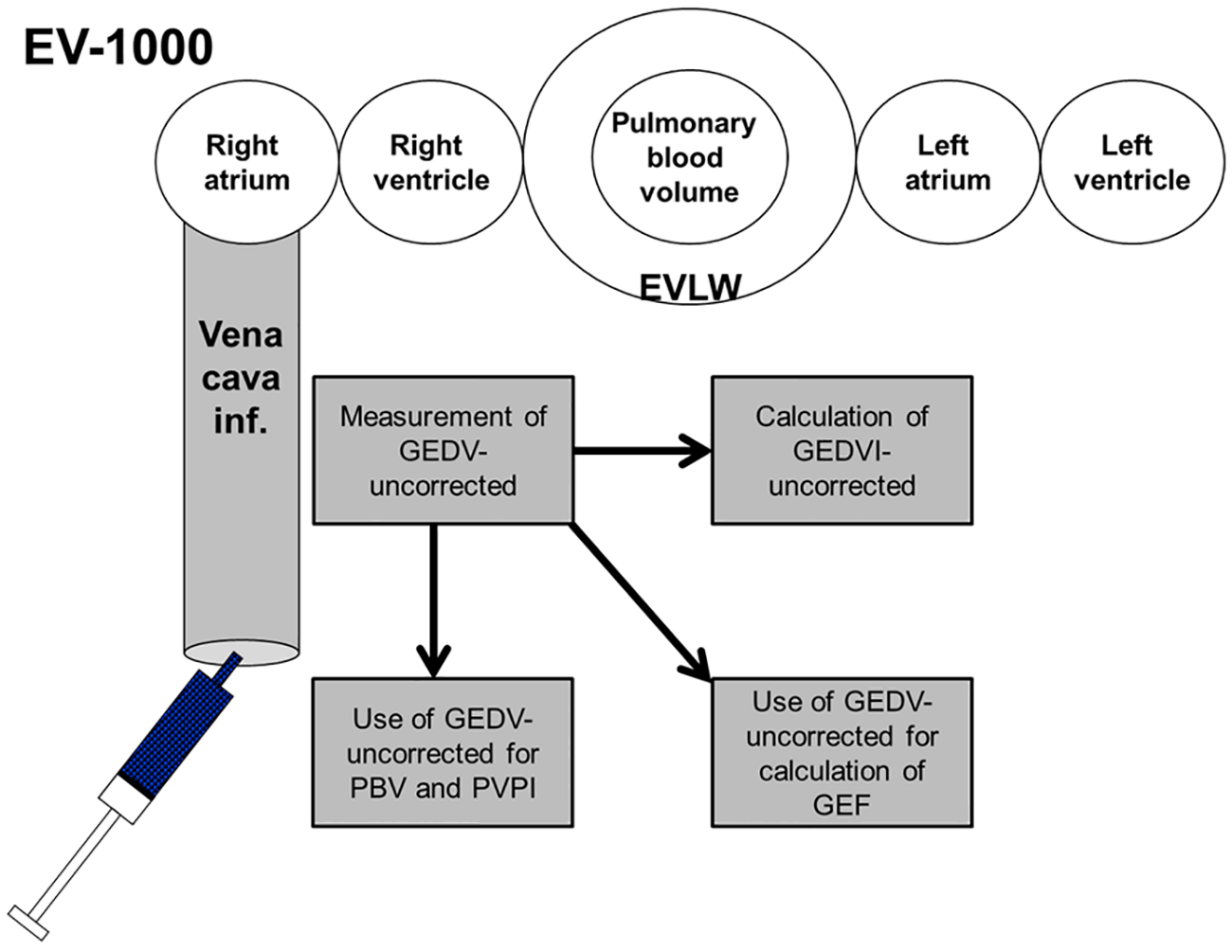
Algorithm assumed for the calculation of GEDV, GEDVI, PBV, PVPI and GEF as displayed by the EV-1000 [5]. Confounders (volume of VCI) and confounded values with marked deviation deviation from corresponding measurements using jugular access (PBV, PVPI_fem) are shaded. The EV-1000 does not correct any parameter derived from femoral CVC indicator injection. GEDV(I): global end-diastolic volume (index) PBV: pulmonary blood volume PVPI: pulmonary vascular permeability index GEF: global ejection fraction

Fig 2 summarizes the present knowledge about correction for *femoral* CVC indicator injection derived TPTD in the latest PiCCO-algorithm [24–27]: The primarily measured GEDV_fem_uncor is indexed to predicted body surface area (BSA) resulting in GEDVI_fem_uncor. In a next step GEDVI_fem_cor is derived from the correction of GEDVI_fem_uncor according to the previously suggested formula. Unindexed GEDV_fem_cor results from multiplication of GEDVI_fem_cor by predicted BSA, and it is used for calculation of GEF_fem_cor. By contrast, PVPI_fem is based on GEDV_fem_uncor irrespective of giving the information about the femoral CVC-position. While the last PiCCO-algorithm at least corrects GEDVI_fem and GEF_fem, to the best of our knowledge the EV-1000 does not correct any parameter derived from femoral CVC indicator injection (Fig 3; [5]).

### Limitations of the study

This study is a single centre study including patients from a general ICU with predominantly medical patients. With a total of 54 measurements performed in 25 patients the number of measurements per patient ranged from 1 to 4 (mean 2.2). Nevertheless, the results derived from the totality of measurements were confirmed by the analyses of the first measurement in each patient. However, larger and multi-centric trials are still required to improve the correction formula suggested for GEDVI derived from 24 patients [24] and to establish a consistent calculation of all TPTD-derived parameters.

## Conclusions

Femoral indicator injection for TPTD results in significantly lower values for PVPI and GEF. While the last PiCCO algorithm appropriately corrects for GEF, this correction obviously is not applied to PVPI. Therefore, GEF-values can be used in case of femoral CVC, but PVPI-values are substantially underestimated. Application of the correction for femoral CVC should be also applied to PVPI.

## Supporting information

S1 Fig Boxplots plots comparing pulmonary vascular permeability index PVPI derived from jugular indicator injection (PVPI_jug), from femoral injection without activating a potential correction by the device (PVPI_fem_uncor), from femoral injection with activating a potential correction by the device (PVPI_fem_cor) and from femoral injection without activating a potential correction by the device, but correcting by the previously suggested formula (PVPI_fem_uncor_form). PVPI_fem_uncor_form was corrected using the formula suggested for correction of femoral indicator injection derived GEDVI: GEDVI_corrected_ [mL / m^2^] = 0.539 * GEDVI_uncorrected_—15.17 + 24.49 * CI_uncorrected_ 2.311* BW_ideal_. PVPI_fem_uncor_form was calculated by multiplying PVPI_fem_uncor with the ratio 0.25*GEDV_uncorrected_/0.25*GEDV_corrected_.
(TIF)

S2 Fig Boxplots plots comparing global ejection fraction derived from jugular indicator injection (GEF_jug), from femoral injection without activating a potential correction by the device (GEF_fem_uncor) and from femoral injection with activating a potential correction by the device (GEF_fem_cor). (TIF)
