# Supplementary figures and images for: Comparison of pulmonary vascular permeability index PVPI and global ejection fraction GEF derived from jugular and femoral indicator injection using the PiCCO-2 device: A prospective observational study
Source: PLoS One. 2017 Oct 17;12(10):e0178372. doi: 10.1371/journal.pone.0178372 (PMC5644983; doi:10.1371/journal.pone.0178372)

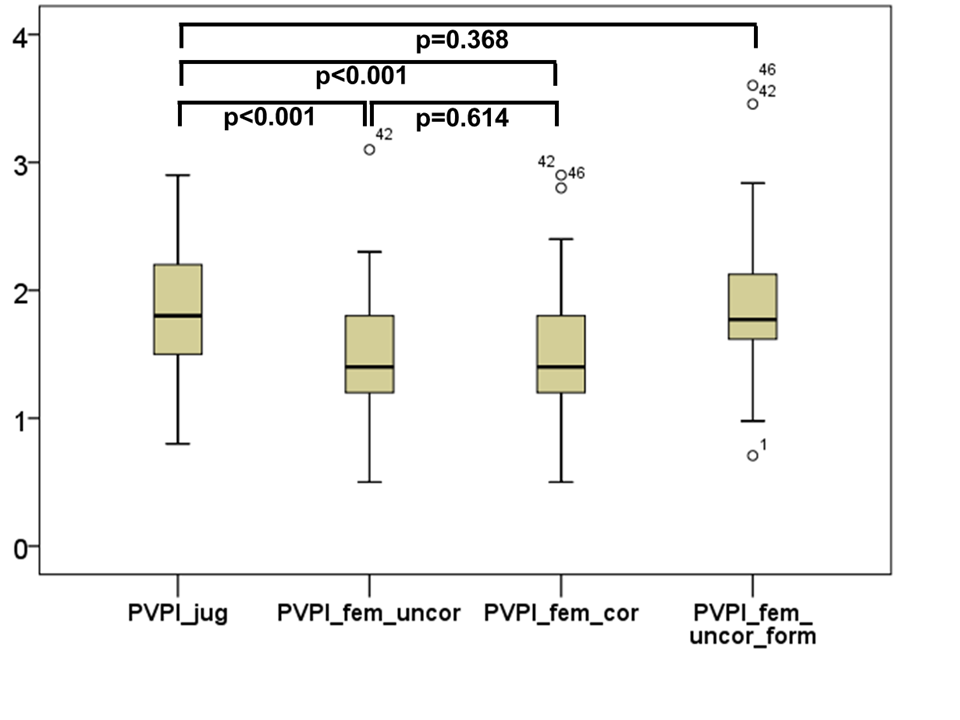

Supplement: S1 Fig — PVPI_fem_uncor_form was corrected using the formula suggested for correction of femoral indicator injection derived GEDVI: GEDVIcorrected [mL / m2] = 0.539 * GEDVIuncorrected—15.17 + 24.49 * CIuncorrected 2.311* BWideal. PVPI_fem_uncor_form was calculated by multiplying PVPI_fem_uncor with the ratio 0.25*GEDVuncorrected/0.25*GEDVcorrected. (TIF) [file pone.0178372.s001.tif]

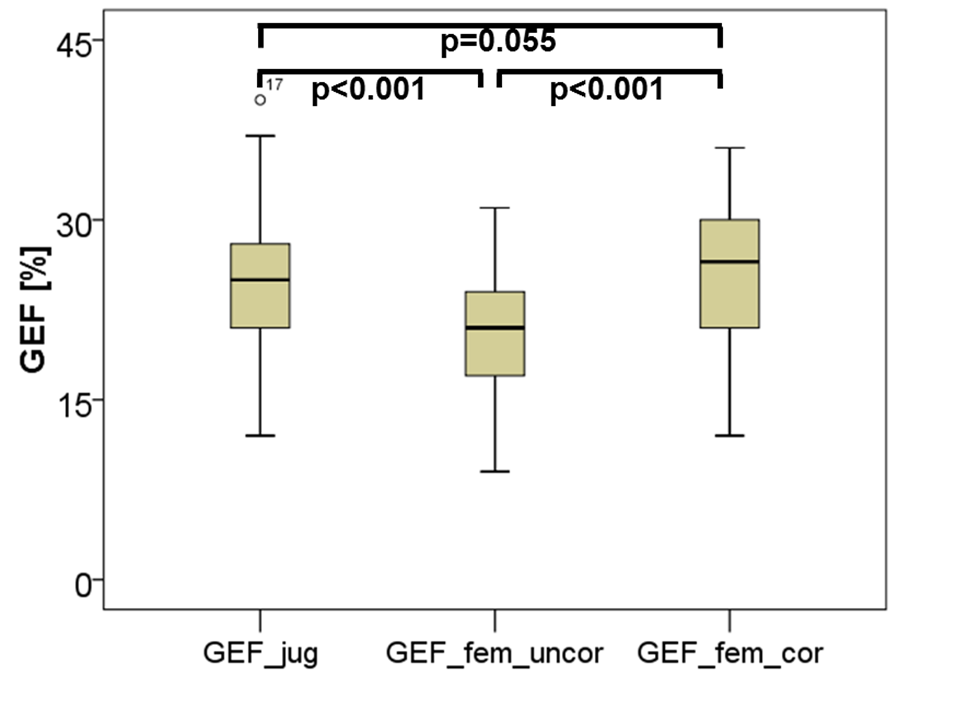

Supplement: S2 Fig — (TIF) [file pone.0178372.s002.tif]
